# Association between antenatal care visits and under-five mortality: An Analysis of the Pakistan demographic and health surveys

**DOI:** 10.1371/journal.pone.0318668

**Published:** 2025-04-11

**Authors:** Rashed Nawaz, Shaoqing Gong, Yaxin Zhao, Neelum Khalid, Zhongliang Zhou, Muhammad Waseem

**Affiliations:** 1 School of Public Health and Health Nutrition, Luohe Medical College, Luohe, Henan Province, China; 2 College of Humanities and Social Development, Northwest Agriculture and Forestry University, Xinyang, Shaanxi, China; 3 School of Public Policy and Administration, Xi’an Jiaotong University, Xi’an, PR China; 4 School of Public Policy and Administration, Hazara University, Mansehra, Pakistan; King Faisal University, SAUDI ARABIA

## Abstract

**Background:**

Pakistan has the third highest under-five mortality rate globally and minimal progress in reducing it, obstructing its achievement of Sustainable Development Goals (SDGs) 3.2 targets. Despite a decline, Pakistan remains one of the top ten contributors to worldwide under-five mortality. Proper antenatal care (ANC) is vital for improving maternal and child health outcomes. This study aims to assess the association between ANC visits and under-five mortality. Moreover, this study also measures the association between the timing of ANC visits and under-five mortality.

**Methods:**

A difference-in-difference (DID) study design with propensity score matching (PSM) was employed to investigate the contributing impact of ANC on the estimation of under-five mortality rates. Statistics from two datasets, 2012 and 2018, of Pakistan demographic and health surveys (PDHS) have been utilized for analyses. This research sample consists of 15698 women aged 15–49 who attended ANC visits at varying times before childbirth.

**Results:**

This study has reported 730 cases of under-five mortality. 98% involved Women who had less than eight or more ANC visits, whereas 2% of under-five mortality cases occurred among those women who had eight or more ANC visits. Overall, our results showed that women who had 1–3 ANC visits reduced the likelihood of under-five mortality in Pakistan by 36% (CI=0.459–0.909, P-value 0.012), the women who had 4–7 ANC visits decreased the likelihood of under-five mortality by 45% (CI=0.364–0.843, P-value 0.006). Moreover, the females with eight or more ANC visits also reduced the likelihood of under-five mortality by 98% (CI=0.512–1.896, P-value 0.966).

**Conclusions:**

Policies and programs focusing more on ANC visits should be implemented to reduce under-five mortality rates significantly. By emphasizing timely and frequent ANC visits, these insights support the development of targeted interventions that can significantly improve child survival rates and support the achievement of SDG targets. Moreover, based on our DID analysis, the implementation of the free maternal and child healthcare (MCH) policy in Pakistan has led to a significant increase in ANC utilization and a consistent reduction in the under-five mortality rates.

## Introduction

Child mortality is a critical global public health indicator, often reflecting the quality of ANC, childbirth, early postnatal care (PNC), and child healthcare outcomes, all of which contribute to a nation’s development status [1–4]. The World Health Organization (WHO) has implemented various strategies to minimize child mortality and improve child health outcomes. Globally, the first six days of a newborn’s life present the highest risk of child mortality, accounting for 15% of under-five mortality. This highlights the importance of proper ANC, safe delivery, and quality PNC. In 2015, approximately 2.6 million neonatal deaths were reported, a 42% reduction since 1990 [5]. Implementing comprehensive health initiatives like the Millennium Development Goals (MDGs), which focus on key aspects of reducing child mortality and improving healthcare outcomes, is vital in reducing child-related illnesses and their effect on mortality rates [4–7]. Despite all these developments, child mortality remains an alarming concern in resource-constraint regions like Africa and Southeast Asia nations, including Pakistan [4].

Pakistan has been progressively dropping behind its South Asian neighbors in reducing maternal and under-five deaths [8,9]. Since the 1990s, neonatal and under-five mortality rates in Pakistan have increased, while progress in reducing maternal mortality has been exceptionally slow [4,10]. Previous studies have identified financial and social barriers [11–13] and poor service quality as key factors obstructing Pakistan’s development in improving maternal and child healthcare outcomes [14–17]. According to the estimate, one in every 20 children goes to death before reaching his fifth birthday in Pakistan [18]. This poses a significant challenge for the government in achieving the SDGs. The SDGs (3.2) aim to reduce neonatal mortality to fewer than 15 deaths per 1,000 births and under-five mortality to at least 25 deaths per 1,000 births by 2030 [11]. The government of Pakistan has committed to reducing neonatal mortality to 12 deaths per 1000 births and under-five mortality to 25 deaths per 1000 live births. ANC is a parental well-being program delivered by qualified healthcare providers and to pregnant women, primarily aimed at identifying and monitoring those high-risk [19–21].

Interactions with professional healthcare providers during ANC visits help to identify and address early health issues, including infectious diseases regarding pregnancy [22]. Studies have shown that none of the countries have successfully reduced maternal mortality rates to fewer than 100 per 100,000 live births without ensuring that every woman receives care from a qualified healthcare provider during the pregnancy and immediately after the delivery [23].

In 2016, the WHO recommended a minimum of eight ANC visits: the first ANC visit should occur within the first three months of pregnancy, two more visits in the second trimester, and the final five visits during the third trimester [24,25]. This research extends these guidelines, emphasizing the number of ANC visits, the timing of the first ANC visit, and the quality of care provided by the healthcare providers.

Pakistan has made some progress in utilizing ANC services, but it remains far from achieving its MGD-5 targets. Many women still give birth without accessing these essential services [26]. In 2013–2014, only 44% of women in Pakistan utilized ANC services, particularly in rural areas [27]. To improve ANC services utilization, reduce maternal mortality, and enhance child health, the government of Pakistan introduced a new cadre in 2006 to provide timely maternal healthcare [28,29]. However, previous studies suggest that community health workers (CMWs) have yet to become significant contributors to ANC service delivery [30].

Numerous researchers have demonstrated that ANC services positively impact the reduction of child mortality [31–33]. Moreover, ANC’s influence on under-five mortality is carefully connected to socio-economic and environmental factors [34]. The importance of ANC visits for maternal and child healthcare cannot be overstated. Nevertheless, prior studies have often been limited to exploring the association between ANC and child mortality. Therefore, this study is designed to fill this gap by investigating the effect of ANC visits on Pakistan healthcare outcomes from 2012 to 2018, concentrating its impact on under-five mortality. The study aims to determine whether the number of ANC visits and the timing of the first visit influence child health outcomes, using a matching approach combined with a DID research design to address confounding variables related to under-five mortality and ANC visits. The outcomes of this study will help policymakers, healthcare providers, and public health organizations upgrade and implement health strategies and interventions to strengthen MCH and achieve SDGs.

## Methods

### Research setting and data

This research utilized the data from the PDHS conducted in 2012 and 2018. The PDHS are national surveys carried out every five years, designed to provide representative data on various health, socio-economic, and demographic factors across all regions of Pakistan, aiming to enhance maternal and child health outcomes. The fourth and fifth wave were utilized in this study have been launched by the National Institute of Population Studies (NIPS) in collaboration with the United States Agency for International Development (USAID) funding [9]. The 2012 and 2018 surveys employed a stratified, two-stage cluster sampling method to select the respondents. Additional details regarding the survey and collection of methods can be found in the final reports of PDHS 2012 and 2018 [35,36]. The data was gathered through interviews with females aged between 15–49 years. The PDHS includes three main questionnaires: household, women, and men. Our research gathered data on child mortality and related covariates from the women’s questionnaire [35,36]. To minimize recall bias, this study utilizes PDHS data, which follow standardized data collection protocols, including pre-tested, structured questionnaires. The recall period for key variables, such as antenatal care visits and the timing of the first visit, is limited to five years or less. Additionally, the PDHS dataset undergoes rigorous data cleaning and validation procedures to identify and correct inconsistencies in self-reported information. These measures help to ensure the reliability and accuracy of the data. The analysis focused on a combined sample of 7461 children from the 2012 survey and 8281 children from the 2018 survey.

### Sample selection

**Fig 1:**
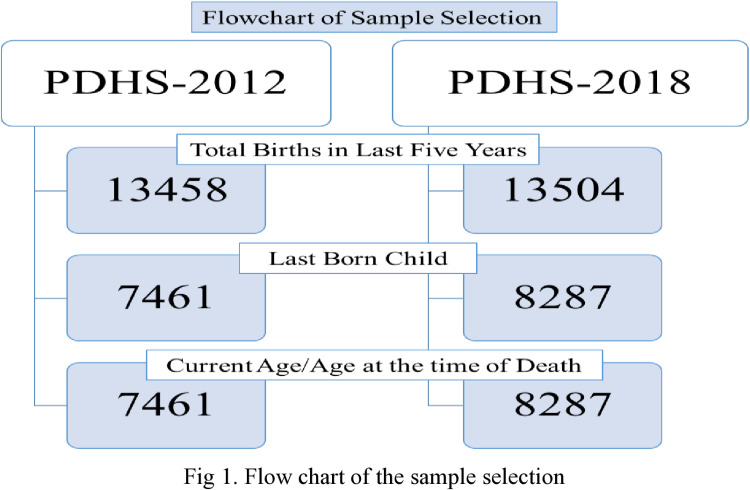
Flow chart of the sample selection.

The sample selection specifically focused on children for whom complete information regarding their survival status was available. Children of mothers who were separated, widowed, not cohabitating, or not in a marital union were excluded from the analysis. This exclusion was made because the father’s involvement in childcare might be limited or absent, particularly in cases where the mother was widowed or single. Additionally, mothers who had been married more than once were excluded to avoid potential residual effects from previous unions on the current one. Three main selection criteria were employed to conduct the analysis. Firstly, it includes births within the five years preceding the survey date. Secondly, during the survey, antenatal and post-natal care information critical to assessing MCH was recorded for last-born children only. Therefore, the sample was further restricted to the last-born children. Lastly, only children with their current age or age at the time of death (recorded in months/years) were included to generate the survival variable. As a result, the sample size was reduced to 15698 out of 100,120 across both surveys. The analysis of under-five mortality included all 15698 children (2012=7461; 2018= 8281). The sample size for the analysis varied slightly across models, depending on the number of missing values among covariates, with variations up to 5.4%. Before executing the analysis, a multicollinearity analysis was conducted to recognize the probable concerns. No concern about the multicollinearity was highlighted, and the variance inflation factors (VIF) have also been less than 5, with an average score of 1.89. Fig 1 depicts the flowchart of the sample selection of the current research.

### Treatment and the outcome variable

In our study, the health outcome is under-five child mortality, meaning the death of a child before the age of five. This information was obtained from the complete birth histories recalled by the interviewed women and recorded in the surveys. We define “treatment” as the use of ANC. Women with 1–3 ANC visits are named treatment 1; they are compared to women who had less than three and no ANC visits (Control 1) during their complete gestation period. The women who had 4–7 ANC visits are considered treatment 2, compared with those who had less than seven or no ANC visits (Control 2). Treatment 3 is for those women who have eight or more ANC visits throughout their entire pregnancy compared with those who have less than eight or no ANC visits (Control 3). The timing of the first ANC visit was analyzed by comparing women who had their first visit during the third trimester (Treatment 4) and those who did not have any ANC visits (Control 4). Treatment 5 consisted of women who had their first ANC visit during the second trimester, compared to women who had their first visit either in the third trimester or no ANC visit (Control 5). Lastly, Treatment 6 included women who had their first ANC visit in the first trimester, compared to those who had their first visit in the second, third, and no ANC visit. The mortality rates for the control groups under five years from 2012 to 2018 may differ due to various unknown factors. The same factors and differences will influence the variation in these rates for the treatment groups in the timing and frequency of ANC visits.

**Fig 2:**
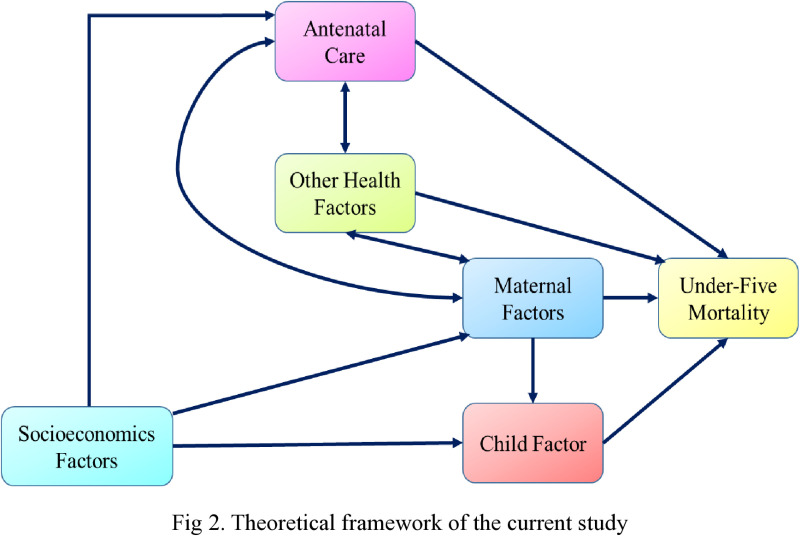
Theoretical Framework of the current study.

The treatment (ANC) is associated with numerous factors that contribute to under-five mortality. Fig 2 demonstrates the association between ANC and the outcome of interest. Socio-economic factors like education level, employment status, and wealth index are all connected with ANC. Furthermore, health-related factors such as place of delivery (POD) also interact with ANC in influencing under-five mortality. These health factors are additionally linked to maternal factors like the mother’s age and age at first birth, which also play a role in the outcome. Child-related factors, including birthweight, birth order, and the preceding interval between births, are connected with socioeconomic and maternal factors. The community-level covariate includes rural/urban residency, all linked to ANC and under-five mortality.

### Ethical considerations

This study involves human participants and procedures and questionnaires for standard DHS surveys have been reviewed and approved by the ICF Institutional Review Board (IRB). Additionally, country-specific DHS survey protocols are reviewed by the ICF, IRB and typically by an IRB in the host country. ICF and IRB ensures that the survey complies with the US Department of Health and Human Services regulations for protecting human subjects (45 CFR 46), while the host country IRB ensures that the survey complies with the laws and norms of the nation. Participants gave informed consent to participate in the study before taking part. Patients and/or the public were not involved in this research’s design, conduct, reporting, or dissemination plans. The DHS also employs culturally sensitive methodologies to ensure that survey instruments are appropriately adapted to local contexts. This includes translating questionnaires into local languages, training interviewers to be culturally aware, and using locally trusted community members to conduct interviews. These measures help overcome cultural barriers and ensure that participants feel comfortable and willing to provide accurate information.

### Statistical analysis

We employed the Cox proportional hazards regression model to determine the direct associations between the independent and dependent variables. This model allows for a focused analysis of the direct effects of ANC visits on the mortality of those under five while accounting for potential confounding factors. Cox proportional hazards regression also enables the insertion of censored data and effectively models time-to-event data with censoring as a dependent variable [37,38].

The analysis involves three models. Model 1 included the outcome variable without including the covariates, while Model 2 introduced additional covariates along with the outcome variable. Moreover, Model 3 enlightened the interaction between the dependent variable and household wealth. This approach is rooted in the Andersen behavioral model of health service utilization [39], which suggests that family wealth can influence factors like access to quality healthcare and nutrition, which are key determinants of childhood mortality [40,41].

We employed a two-stage research design to enhance the comparison among the treated and control groups. Our analysis started by matching individuals with identical household features to ensure equal probability of ANC utilization across both groups. We utilized PSM for this purpose. PSM is a statistical method designed to address the primary drawback of causal inferences in observational studies, where standardized control groups are not established [42]. This technique creates matched pairs of control and treatment individuals with similar propensity scores [43]. Once the matched sample is created, the treatment effect can be directly assessed by comparing outcomes between the treated and control groups [44].

The PSM method was employed to compare under-five mortality between women who utilize ANC and those who do not [45]. PSM was selected because ANC utilization is not random and is significantly influenced by observable and unobservable factors. The variables for PSM were selected based on demographic and socioeconomic covariates from the PDHS that showed a significant association with ANC utilization and child mortality. Using a logistic regression model, women who utilized ANC were matched to those who did not. To assess the balance of covariates before and after PSM, we applied a Chi-Square test, considering a 5% level of significance or higher indicative of imbalance.

We also applied the DID technique to examine the impact of ANC services on under-five child mortality. DID is a quasi-experimental method that compares changes in outcomes over time between an intervention (treatment group) and a non-intervention group (control group) [46]. Although DID is primarily used with panel data to estimate the effects of policies or programs, it has also been employed with repeated cross-sectional data from the same areas, as seen in previous studies [47–49]. We conducted the multivariate normal (MVN) imputation procedure to report the missing values, assuming that the missing data was randomly scattered. We have run the number of imputations to 20. MVN imputation was selected because of its capability to handle the complex interdependencies among the variables. We ensured that the imputed values accurately predicted the missing data, and there was no significant difference in the results before and after imputation [50]. All the analyses were performed in STATA software 16.0 (Stata Corp, College Station, TX, USA) at a 95% confidence interval.

The covariates included were the child’s gender, birth order, preceding birth interval, and perceived birthweight. Maternal factors include the mother’s age (in years), education level (measured continuously), number of living children (constantly measured), Caesarean section history (No=0, Yes=1), multiple births (singleton=0, multiple=1), father’s education (measured continuously), and employment status in the year before the survey. Socioeconomic factors include delivery in a health facility (non-medical=0, medical=1) and family wealth. Community-level factors include residence status (Urban=0, Rural=1). Detailed information is provided in Table 1.

**Table 1:** Description and operationalization of variables.

Variables	Description	Coding	Type	Mini- mum	Maxi- mum
**Outcome Variable**					
Under-five mortality	The probability of a newborn dying between the ages of 0 and 5 Years of age	Failure=0; Event=1	Time-to-Event	0	1
**Treatment Variable**					
Antenatal care (ANC)	Antenatal care		Continuous	0	99
**Control variables at the maternal and child factors**					
Sex	Sex of the child	Male=1 Female=2	Nominal	1	2
Birth order	The birth order of the child		Continuous	1	17
Birthweight	weight of the child at birth as perceived by the mother.	Small=1; Average=2 Large=3	Ordinal	1	3
**Control variables at the maternal factors**					
Age of mother’s	Age of mother in years		Continuous	15	49
Mother’s education	No. of years of formal education acquired		Continuous	0	16
Parity	No living children		Continuous	0	14
Working status	Whether or not the mother has been employed in a paid job in the preceding 12 months	Yes=2; No=1	Nominal	1	2
Father education	No of years of formal education acquired		Continuous	0	16
Place of delivery	Whether the child is born in a medical facility or not	Non-Medical=0 Medical =1	Nominal	0	1
Birth interval	Time space between the current birth and the preceding birth	0=, <24; 1= >24	Binary	0	1
Family wealth index	The wealth index, based on the ownership of certain assets in a household		continuous	-0.13	0.95
**Control variables at the community level**					
Residence status	Whether the location of residence is rural or urban	Urban=0; Rural=1	Nominal	0	1

Note: WHO= World Health Organization; ANC= Antenatal Care;; POD= Place of Delivery; Based on WHO guidelines

## Results

### Socio-demographic characteristics of the sample

Table 2 presents the socio-demographic characteristics of the pooled sample. This study included 13,458 children from PDHS 2012 and 13,504 children from PDHS 2018, all born within five years before each survey. Of these, 7,461 children (2012) and 8,287 (2018) met the selection criteria. Among the sample, 382 (5.12%) children in 2012 and 348 (4.20%) in 2018 died within five years. The gender distribution was nearly equal in both surveys, with 51.41% male in 2012 and 52.02% male in 2018. The average age of the mothers was 29.6 years, with a standard deviation of 6.34 years in both surveys. Approximately 56% (2012) and 54% (2018) of households were rural, with average family wealth indices of -0.13 (SD = 0.95) and -0.09 (SD = 0.98), indicating prevalent poverty. The ANC composite scores were 1.25 (SD = 0.97) and 1.46 (SD = 0.89) in 2012 and 2018 respectively.

**Table 2:** Weighted percentage of distribution of sample characteristics.

Variable	2012				2018				
	**N**	**%**	**Mean**	**SD**	**N**	**%**	**Mean**	**SD**	
**Outcome variable**									
Under-five mortality									
No	7079	94.88			7939	95.80			
Yes	382	5.12			348	4.20			
**Treatment Variable**									
Antenatal care			1.25	0.97				1.46	0.89
Child sex									
Male	3836	51.41			4311	52.02			
Female	3625	48.59			3976	47.98			
**Birth order**									
First born	1389	18.62			1586	19.14			
2-3 born	2683	35.96			3240	39.10			
4^th^ born	3389	45.2			3461	41.76			
Birth interval (< 24 months)	1844	24.78			1966	23.79			
24 months or more	5598	75.22			6299	76.21			
**Perceived birth weight**									
Small	1488	20.01			1567	18.98			
Average	5433	73.06			6038	73.15			
Large	515	6.93			649	7.86			
**Type of birth**									
Singleton	7374	98.83			8159	98.46			
Multiple births	87	1.17			128	1.54			
**Caesarian session**									
No	6433	86.38			6655	80.37			
Yes	1014	13.62			1625	19.63			
**Place of delivery**									
Non-medical	3512	47.12			2742	33.14			
Medical	3942	52.88			5531	66.86			
Mother’s Age (in years)			29.58	6.42				29.56	6.34
Mother education (in years)			3.88	5.01				4.51	5.28
Mother’s employment									
Not employed	6083	81.67						7316	88.29
Employed	1365	18.33						970	11.71
Parity			3.40	2.16				3.25	2.06
Wealth index			-0.13	0.95				-0.09	0.98
Father’s education (in years)			6.63	5.36				7.02	5.22
**Residence status**									
Urban	3278	43.94			3738	45.11			
Rural	4183	56.06			4549	54.89			
Observations	† N < 13458					† N < 13504			

Note: SD= Standard Deviation; ANC= Antenatal Care;

POD= Place of Delivery; Based on WHO guidelines;

†† Number of observations less than 13548 & 13504.

Table 3 illustrates the mortality rates of those under five based on the timing of the first ANC visit. For women who had no ANC visit, the under-five mortality rate was 4.44% and 12.50% in 2012 and 2018, respectively. Similarly, for the women who had their first ANC visit in the first trimester, the mortality rate reduced from 4.08% in 2012 to 3.63% in 2018. Furthermore, the Women who had their first ANC visit in the second trimester experienced a decline in under-five mortality to 4.27% in 2012 and 3.79% in 2018. Finally, for those women who had their first ANC visit in the third trimester, the mortality rate increased to 5.36% in 2012, and in 2018, the mortality rate decreased to 5.78%.

**Table 3:** Under-five mortality rates for timing of first ANC visits.

	2012		2018	
	Under-five mortality		Under-five mortality	
	Yes	No	Yes	No
No visit	2 (4.44)	43 (95.56)	2 (12.50)	14 (87.50)
First trimester	130 (4.08)	3060 (95.92)	159 (3.63)	4224 (96.37)
Second trimester	64(4.27)	1434 (95.73)	75 (3.79)	1904 (96.21)
Third trimester	43 (5.36)	759 (94.64)	36 (5.78)	587 (94.22)

### Multivariate analysis

The key supposition of Cox regression is proportional hazards, which we verified using the scaled Schoenfeld residuals test [37]. The test outcomes disclosed no violation of this assumption, as the proportionality for the overall model, as well as for each predictor and covariate, was not significant (p-values > 0.05).

### The association between the number of ANC visits and child mortality

The results of the unadjusted model are presented in Table 4. They highlight insignificant relationships between the number of ANC visits and child mortality between the three models across the 2012 and 2018 PDHS surveys, except for the number of ANC visits 1–3 in model 2 for PDHS 2012, which showed a reduction in child mortality by (HR=0.86; CI=0.47–0.97; p > 0.05). Socio-demographic factors linked to an increased risk of child mortality include higher multiple births, older maternal age, mother education, and mother employment. Conversely, factors like female gender, birth intervals, higher birthweight, place of delivery, and higher parity are associated with a protective effect.

**Table 4:** Cox proportional regression models of factors associated with ANC and child mortality.

Variables	2012					2018						
**Model 1**		**Model 2**		**Model 3**		**Model 1**		**Model 2**		**Model 3**	
**CR**	**SE**	**HR**	**SE**	**HR**	**SE**	**CR**	**SE**	**HR**	**SE**	**HR**	**SE**
ANC ref (=0)												
1-3 ANC Visits	0.76	(0.13)	0.68*	(0.12)	0.83	(0.14)	1.18	(0.29)	1.13	(0.27)	1.05	(0.23)
4-7 ANC Visits	0.76	(0.15)	0.68	(0.18)	0.95	(0.24)	0.67	(0.17)	0.68	(0.17)	0.63	(0.16)
8 or more ANC Visits	0.77	(0.18)	0.70	(0.21)	1.29	(0.39)	0.91	(0.28)	0.96	(0.29)	0.93	(0.31)
Female (ref=male)			1.13	(0.16)	1.18	(0.17)			0.95	(0.13)	0.97	(0.13)
Ref= Birth interval (<24 months)			1.00	(0.00)	1.00	(0.00)			1.00	(0.00)	1.00	(0.00)
24month or more			0.50***	(0.07)	0.41***	(0.06)			0.49***	(0.08)	0.36***	(0.06)
Perceived birth weight												
(ref=small)			1.00	(0.00)	1.00	(0.00)			1.00	(0.00)	1.00	(0.00)
Average			0.72*	(0.11)	0.78	(0.11)			0.86	(0.16)	0.88	(0.16)
Large			0.99	(0.27)	0.99	(0.25)			0.68	(0.21)	0.68	(0.21)
Caesarian session (ref=No)			1.00	(0.00)	1.00	(0.00)			1.00	0.00	1.00	0.00
Yes			0.94	(0.19)	0.93	(0.17)			1.00	(0.19)	0.93	(0.16)
Place of delivery												
Medical (ref= non-medical)			1.37	(0.28)	1.47*	(0.28)			1.25	(0.24)	1.19	(0.23)
Multiple births (ref=singleton)			4.22***	(1.33)	6.11***	(1.63)			3.78***	(1.42)	5.97***	(1.55)
Mother’s Age (in years)					1.11***	(0.01)					1.09***	(0.01)
Mother education (in years)					0.93**	(0.02)					0.92***	(0.02)
Mother employment (ref=No)					1.44**	(0.19)					1.53*	(0.28)
Parity					0.61***	(0.03)					0.47***	(0.04)
Family wealth index					0.85	(0.11)					0.90	(0.11)
Father’s education (in years)					0.98	(0.02)					1.03	(0.02)
Rural (ref= urban)					1.24	(0.25)					0.98	(0.18)
Observations	7,443		7,385		7,363		6,773		6,709		6,599	

Analyses are clustered at the household level; CR= Crude ratio; HR= Hazard ratio; ref. = Reference Group;

***p<0.001,

**p<0.01,

*p<0.05 Model 1; without covariates; Model 2 adjusted for covariates; Model 3; added family wealth index with covariate

### Propensity score matching results

The PSM generated a matched sample of 4,817 birth cases. Table 5 compares the statistics for individual demographic and socioeconomic characteristics of women who had ANC visits with those who did not before and after matching. Before PSM, no baseline characteristics showed a significant difference (P-value < 0.05) in under-five mortality between the treatment and control groups. After the PSM, all characteristics showed a significant difference (P-value > 0.05) in under-five mortality between the treatment and control groups. This indicates that the PSM approach effectively minimized the differences in observed characteristics between the groups. Fig S1 explains ANC 1–3 visits for match and unmatched observations. Additionally, Fig S2 demonstrates number of ANC 4–7 visits before and after matching and finally, Fig S3 illustrates the number of ANC visits eight or more for match and unmatched observations.

**Table 5:** Statistics for individual and socioeconomic characteristics before and after PSM.

Variables	ANC before matching			ANC after matching		
**Total Observations**	**N=4584**			**N=2804**		
**Treatment 1**	Treated	Control	*p-*value	Treated	Control	*p-*value
Mother age	29.142	30.869	0.000	29.142	28.851	0.103
Maternal education	2.829	0.884	0.000	2.829	3.037	0.075
Parity	3.481	4.219	0.000	3.481	3.429	0.383
Wealth index	-0.302	-0.760	0.000	-0.302	-0.310	0.736
Employed	1.200	1.210	0.386	1.200	1.201	0.945
Father education	5.998	4.119	0.000	5.998	5.847	0.278
Residence	1.628	1.729	0.000	1.628	5.847	0.279
Gender	1.492	1.496	0.814	1.492	1.477	0.284
Birth weight	1.850	1.827	0.119	1.850	1.874	0.078
Preceding birth interval	1.735	1.712	0.087	1.735	1.733	0.877
Twins	0.009	0.005	0.182	0.009	0.009	0.886
Cesarian	0.080	0.013	0.000	0.080	0.059	0.002
Place of delivery	0.495	0.154	0.000	0.495	0.485	0.476
**Treatment 2**						
**Total observations**	**N=3832**			**N=1477**		
Mother age	29.142	30.869	0.000	29.142	28.851	0.103
Maternal education	5.963	0.884	0.000	5.963	6.039	0.647
Parity	2.926	4.222	0.000	2.926	2.675	0.000
Wealth index	0.265	-0.760	0.000	0.265	0.309	0.160
Employed	1.200	1.210	0.386	1.200	1.201	0.945
Father education	8.376	4.122	0.000	8.376	7.845	0.001
Residence	1.451	1.729	0.000	1.451	1.928	0.405
Gender	1.492	1.496	0.814	1.492	1.477	0.284
Birth weight	1.903	1.827	0.000	1.903	1.928	0.090
Preceding birth interval	1.780	1.712	0.000	1.780	1.753	0.046
Twins	0.012	0.005	0.029	0.012	0.007	0.148
Cesarian	0.222	0.013	0.000	0.222	0.749	0.000
Place of delivery	0.755	0.154	0.000	0.755	0.755	1.000
**Treatment 3**						
**Total observations**	**N=2834**			**N=536**		
Mother age	29.142	30.869	0.000	29.142	28.851	0.103
Maternal education	8.631	0.884	0.000	8.631	8.205	0.057
Parity	2.541	4.222	0.000	2.541	2.033	0.000
Wealth index	0.771	-0.760	0.000	0.771	0.846	0.075
Employed	1.200	1.210	0.386	1.200	1.201	0.945
Father education	9.907	4.122	0.000	9.907	8.056	0.000
Residence	1.254	1.729	0.000	1.254	1.242	0.554
Gender	1.492	1.496	0.814	1.492	1.477	0.284
Birth weight	1.951	1.827	0.000	1.951	1.964	0.472
Preceding birth interval	1.828	1.712	0.000	1.828	1.848	0.255
Twins	0.012	0.005	0.029	0.012	0.007	0.148
Cesarian	0.361	0.013	0.000	0.361	0.076	0.000
Place of delivery	0.913	0.154	0.000	0.913	0.902	0.421

### The estimation of DID outcomes

Table 6 presents the primary outcome of the DID analysis for the matched sample with all covariates added to the model. The estimates for Treatment 1 ANC visit 1–3 recommended that compared to women who had no ANC visit, the women who had 1–3 ANC visit decreased the likelihood of under-five mortality by 36% (CI = 0.45–0.90%, P-value = 0.012). This estimate is statistically significant, with a P-value of 0.05, indicating a significant difference in under-five mortality between women who had 1–3 ANC visits and those who did not attend ANC at all. Correspondingly, under-five mortality was 45% lower for women with 4–7 ANC visits. This difference was also statistically significant (CI = 0.36–0.8%, P-value = 0.006) compared to women who had 1–3 ANC visits or no ANC visits. Finally, Table 6 demonstrates that women with eight or more ANC visits compared with women who had 4–7 ANC visits, women who had 1–3 ANC visits, and those who did not attend ANC at all also reduced the likelihood of under-five mortality by (OR= 0.98 CI = 0.51–1.89%, P-value < 0.966).

**Table 6:** DID for the number of ANC visits with covariates in all models.

Treatment Effects	(OR)	(SE)	*P-*value	95% C1
**DIDI Treatment 1**				
**ANC 1–3 visits**	0.646**	0.112	0.012	0.459-0.909
Mother age	1.1358***	0.012	0.000	1.111-1.159
Maternal education	0.929***	0.018	0.000	0.893-0.967
Parity	0.577***	0.023	0.000	0.533-0.626
Wealth index	1.016	0.098	0.862	0.841-1.228
Employed	1.397**	0.201	0.020	1.052-1.854
Father education	0.997	0.013	0.842	0.971-1.023
Residence	1.076	0.154	0.605	0.813-1.425
Female	1.210	0.138	0.095	0.967-1.515
Birth weight	0.790**	0.868	0.032	0.637-0.980
Preceding birth interval	0.387***	0.046	0.000	0.305-0.490
Twins	4.586***	1.781	0.000	2.142-9.819
Cesarian	1.476	0.313	0.067	0.973-2.239
Place of delivery	1.438**	0.188	0.006	1.112-1.860
Yes	1.048	0.166	0.765	0.767-1.431
LR test Model fitness	299.10***			
Log Likelihood	-1218.921			
Number of observations	6875			
**DID Treatment 2**				
**ANC 4–7 visits**	0.554**	0.118	0.006	0.364-0.843
Mother age	1.130***	0.014	0.000	1.103-1.158
Maternal education	0.913***	0.018	0.000	0.878-0.949
Parity	0.531***	0.027	0.000	0.480-0.587
Wealth index	0.968	0.103	0.764	0.784-1.194
Employed	1.314	0.236	0.129	0.923-1.869
Father education	1.003	0.015	0.843	0.973-1.033
Residence	0.983	0.156	0.915	0.720-1.342
Female	1.126	0.148	0.366	0.869-1.460
Birth weight	1.009	0.133	0.942	0.778-1.309
Preceding birth interval	0.365***	0.050	0.000	0.278-0.480
Twins	6.901***	2.643	0.000	3.257-14.622
Cesarian	1.046	0.224	0.833	0.687-1.592
Place of delivery	1.099	0.177	0.558	0.800-1.508
Yes	0.909	0.162	0.597	0.640-1.292
LR test Model fitness	266.18***			
Log Likelihood	-921.540			
Number of observations	5655			
**DID Treatment 3**				
**ANC eight and more visits**	0.985	0.329	0.966	0.512-1.896
Mother age	1.132***	0.151	0.000	1.095-1.170
Maternal education	0.907***	0.024	0.000	0.861-0.956
Parity	0.581***	0.037	0.000	0.511-0.660
Wealth index	0.760	0.114	0.070	0.566-1.022
Employed	1.847**	0.421	0.007	1.181-2.888
Father education	1.034	0.219	0.107	0.992-1.078
Residence	0.693	0.147	0.084	0.457-1.050
Female	1.137	0.199	0.462	0.80-1.605
Birth weight	0.946	0.160	0.747	0.679-1.319
Preceding bBirth interval	0.271***	0.051	0.000	0.187-0.392
Twins	5.692**	3.115	0.001	1.946-16.642
Cesarian	0.923	0.256	0.775	0.536-1.590
Place of delivery	1.082	0.246	0.727	0.692-1.692
Yes	0.620*	0.151	0.051	0.384-1.002
LR test Model fitness	147.49***			
Log Likelihood	-513.041			
Number of observations	2782			

Table 7 describes the impact of the timing of the first antenatal care (ANC) visit on under-five mortality. The analysis found no statistically significant effect of a first ANC visit in the third trimester on under-five mortality (P-value = 0.242) compared to women without ANC visits. In contrast, for the women who had their first ANC visit in the second trimester, there was also no statistically significant effect of a first ANC visit in the second trimester on under-five mortality (P-value = 0.106) compared to those women who had their first ANC visit in the third trimester and did not have any ANC visits. Furthermore, the Women who had their first ANC visit in the first trimester experienced a 45% reduction in under-five mortality (CI = 0.332–0.629, P-value < 0.000) compared to those with later visits.

**Table 7:** DID for the timing of first ANC Visits with covariates in all Models.

Treatment Effects	(OR)	(SE)	*P-*value	95% C1
**DIDI Treatment 4**				
**Third Trimester**	0.193	0.271	0.242	0.012-3.029
Mother age	1.211***	0.052	0.000	1.113-1.318
Maternal education	0.853*	0.065	0.065	0.733-0.922
Parity	0.486***	0.092	0.000	0.335-0.705
Wealth index	0.736	0.278	0.419	0.350-1.547
Employed	0.968	0.539	0.954	0.325-2.885
Father education	1.103	0.056	0.056	0.997-1.220
Residence	1.528	0.892	0.468	0.486-4.801
Female	1.167	0.509	0.722	0.497-2.744
Birth weight	0.976	0.388	0.953	0.447-2.130
Preceding birth interval	0.257***	0.126	0.006	0.098-0.673
Twins	19.526*	27.878	0.037	1.189-320.540
Cesarian	1.355	0.886	0.642	0.376-4.881
Place of delivery	1.955	0.961	0.173	0.745-5.127
Yes	5.280	7.205	0.173	0.745-5.127
LR test Model fitness	298.10***			
Log Likelihood	-87.702			
Number of observations	548			
**DID Treatment 5**				
**Second Trimester**	0.549	0.203	0.106	0.265-1.136
Mother age	1.157***	0.284	0.000	1.103-1.214
Maternal education	0.876**	0.348	0.001	0.810-0.947
Parity	0.386***	0.057	0.000	0.288-0.517
Wealth index	0.768	0.161	0.211	0.509-1.160
Employed	1.191	0.402	0.604	0.614-2.309
Father education	1.048	0.306	0.103	0.990-1.110
Residence	1.630	0.551	0.148	0.840-3.164
Female	0.937	0.238	0.799	0.569-1.542
Birth eeight	0.995	0.237	0.986	0.624-1.588
Preceding birth interval	0.210***	0.063	0.000	0.116-0.380
Twins	12.518***	9.669	0.001	2.754-56.890
Cesarian	1.234	0.446	0.559	0.608-2.506
Place of delivery	1.693	0.505	0.077	0.944-3.038
Yes	1.478	0.536	0.280	0.726-3.009
LR test Model fitness	266.18***			
Log Likelihood	-254.887			
Number of observations	1939			
**DID Treatment 6**				
**First Trimester**	0.457***	0.074	0.000	0.332-0.629
Mother age	1.107***	0.010	0.000	1.086-1.129
Maternal education	0.944***	0.013	0.000	0.918-0.970
Parity	0.387***	0.022	0.000	0.44-0.434
Wealth index	0.895	0.072	0.169	0.764-1.048
Employed	1.206	0.167	0.175	0.919-1.582
Father education	0.981	0.011	0.107	0.958-1.004
Residence	1.057	0.127	0.645	0.834-1.340
Female	1.046	0.104	0.645	0.861-1.272
Birth weight	0.794*	0.077	0.018	0.656-0.960
Preceding birth interval	0.232***	0.027	0.000	0.183-0.294
Twins	11.514***	3.056	0.000	6.843-19.374
Cesarian	0.860	0.128	0.315	0.642-1.15
Place of delivery	0.864	0.100	0.213	0.688-1.086
Yes	1.075	0.121	0.518	0.862-1.342
LR test Model fitness	541.44***			
Log Likelihood	-1578.616			
Number of observations	9135			

## Discussion

Our study utilized the data from widespread nationwide representative Pakistan DHS (2012 and 2018) to analyze the association between the number of ANC visits and the time for the first ANC visit on child health outcomes, specifically, under-five mortality in Pakistan. Firstly, we used Cox proportional hazard regression to measure the association between the number of ANC visits and under-five mortality. Though the results are insignificant, the overall results indicated a reduction in under-five mortality and an increase in ANC visits. The ratio of under-five mortality decreased from 5.12% in 2012 to 4.20% in 2018. These patterns of results were consistent across both PDHS waves. Several socio-demographic factors were also connected with an increased risk of child mortality, including higher multiple births, older maternal age, mother education, and mother employment. Conversely, factors like female gender, longer birth intervals, higher birthweight, place of delivery, and higher parity are associated with a protective effect. Our study’s finding is consistent with the finding of [51].

Furthermore, constructed on the DID model estimation, our research outcomes provided proof of a 45% decrease in under-five mortality for the women who had 4–7 ANC visits compared to the women who had 1–3 ANC visits as compared to the women who had no visit at all. The results also highlighted that only 2% of the under-five mortality occurs for the women who had eight or more ANC visits as compared to the women who had 4–7 ANC visits, women who had 1–3 ANC visits as compared to the women who had no visit at all.

To the best of our knowledge, this study is one of the first to specifically examine the impact of the number of ANC visits and the timing of the first ANC visit on under-five child mortality in Pakistan. Studies have shown that increasing ANC visits reduces under-five mortality [52–54]. For instance, Jana Kuhnt and Sebastian Vollmer [55] analyzed data from 193 DHS surveys (1990–2013) and found that a single ANC visit reduced neonatal mortality by 1.04% and infant mortality by 1.07%. Similarly, a study from Bangladesh employing multivariate logistic regression across three DHS surveys found that ANC visits with professional healthcare providers are linked to a decreased likelihood of under-five mortality among females [56]. Another study in Kenya by Malachi et al. [57] also examined the effectiveness of ANC services in reducing neonatal mortality. Using the binary logistics regression, the study found that mothers with ANC visits have lower rates of neonatal mortality. The Government of Pakistan has made significant efforts to improve MCH, offering free services, particularly in remote areas. Drawing on this well-acknowledged evidence, the Pakistani Government announced an innovative squad of skilled birth attendants [58]. Previous studies from Pakistan have highlighted that factors such as place of delivery, education, wealth, and funding for demand-side interventions are linked with the utilization of ANC services [59]. Our research findings will assist policymakers in shaping policies that reflect the impact of ANC visits with professional healthcare providers on reducing under-five mortality. This will also promote the increased utilization of ANC services.

### Strengths and limitations

The main strength of the current study lies in its use of nationwide demographic data. Even though there has been no randomized control experiment, a repetitive cross-sectional examination from the identical sample frame can be utilized to acquire causal interpretation [60]. The double methodology we have engaged in the present research has confirmed that bias has been reduced by comparing the treatment and control groups, which should be identical before applying the quasi-experimental research design for causal inference.

The DID methodology assumes that persons’ unmeasured serious features can affect the result based on the variances amongst the treatment and control groups [61]. It was highlighted earlier in the current research that the utilization of the double approach has minimized possible biases. Still, we cannot omit the unmeasured features. Nevertheless, we have selected our covariates with the help of a cautious understanding of the prior works. The limitation connected with this research is the utilization of cross-sectional data, which prevents establishing a causal relationship. Additionally, cultural factors that discourage discussing the deceased or the emotional distress of recalling a lost child may have led to underreporting of mortality. Another limitation of the current study is the quality of DHS statistics. As irregular to most nationwide investigations, there have been missing values in the statistics. The concern regarding the missing values has been lectured by using multiple imputations. The procedure of the multiple imputations was effective for the statistics missing at random; in addition, the present research safeguarded that the imputed statistics were projections of the missing statistics. The current research also highlighted that no constructive dissimilarities were found before or after the multiple imputations.

### Policy implications

Improving access to ANC should be a priority to reduce under-five mortality. Policymakers should implement programs that promote early and regular ANC visits, particularly in rural areas. This could involve expanding the healthcare structure, training skilled healthcare specialists, providing community-based health services, offering transportation support, and increasing awareness about the importance of early pregnancy check-ups through community awareness campaigns. Our results are helpful for policymakers, healthcare providers, and public health organizations. Aligning national health policies with WHO guidelines, including recommending at least eight ANC visits with the first visit in the first trimester, will improve maternal and child health outcomes. Additionally, investing in quality ANC services and training healthcare providers will be crucial for achieving the SDGs of reducing child mortality by 2030.

## Conclusions

This study employed PSM and DID logistic regression analysis to discover the association between ANC visits and under-five mortality. Our findings suggest that the number of ANC visits and the timing of the first visit significantly reduce under-five mortality. We recommend at least eight ANC visits, with the first visit occurring during the first trimester, by the 2016 WHO guidelines. To make meaningful progress in lowering under-five mortality rates and achieving the SDGs by 2030, it is vital to implement programs that promote free MCH services early and regular ANC visits, especially in remote and rural regions where access to healthcare services is limited. These programs could include community outreach, transportation support, and education about the importance of early prenatal care. Additionally, we recommend future research that explores the causal relationship between ANC and under-five mortality in greater detail and assesses the quality and accessibility of ANC services, as these factors may also play a key role in improving child health outcomes. Addressing barriers to accessing quality care, especially for vulnerable populations, will be crucial to achieving sustainable reductions in under-five mortality.

## Supporting information

S1 Fig ANC visits 1–3 for match and unmatched observations. (TIF)

S2 Fig ANC visits 4–7 for match and unmatched observations. (TIF)

S3 Fig ANC visits eight or more for match and unmatched observations. (TIF)

S4 File 2012+2018.DTA. (DTA)

## Abbreviations

ANC: Antenatal care
DID: Difference-in-Differences
IRB: Institutional Review Board
MCH: Maternal and Child Healthcare
MDGs: Millennium Development Goals
PDHS: Pakistan Demographic and Health Survey
POD: Place of delivery
PSM: Propensity score matching
SDGs: Sustainable development goals
