# Supplementary figures and images for: Association between antenatal care visits and under-five mortality: An Analysis of the Pakistan demographic and health surveys
Source: PLoS One. 2025 Apr 11;20(4):e0318668. doi: 10.1371/journal.pone.0318668 (PMC11990642; doi:10.1371/journal.pone.0318668)

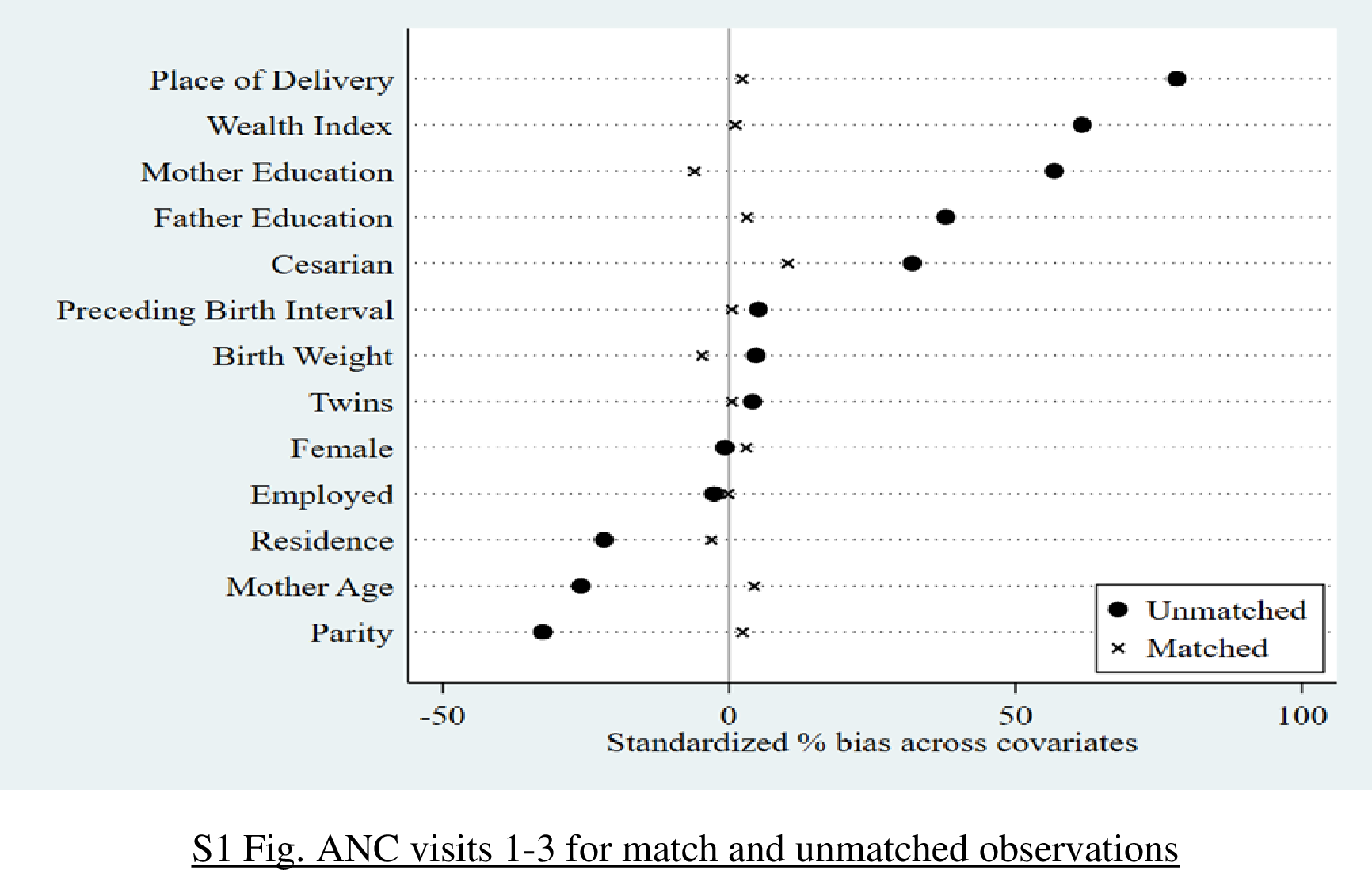

Supplement: S1 Fig — (TIF) [file pone.0318668.s001.tif]

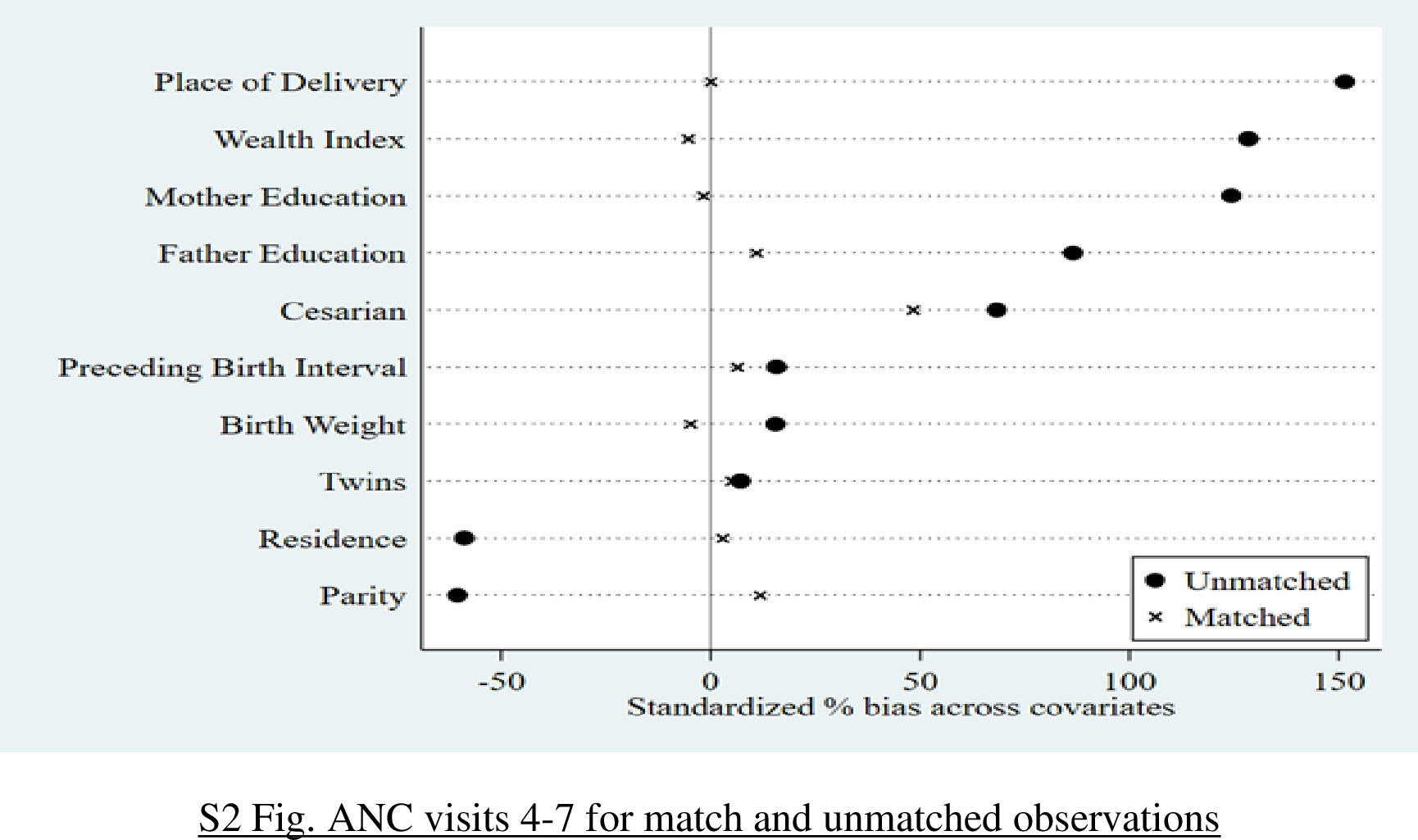

Supplement: S2 Fig — (TIF) [file pone.0318668.s002.tif]

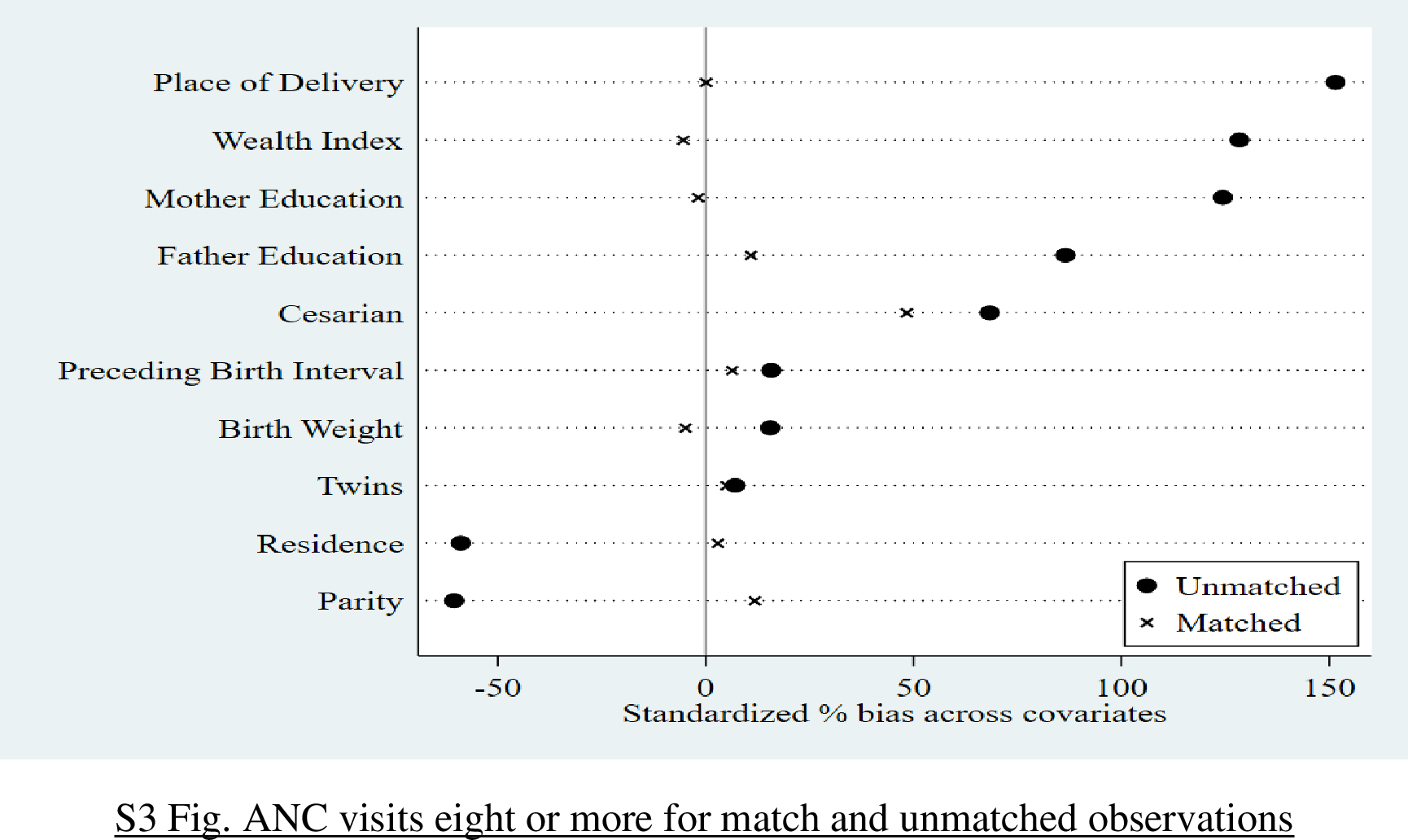

Supplement: S3 Fig — (TIF) [file pone.0318668.s003.tif]
